# Fungal Pigments: More Insights from Colorful Fungi

**DOI:** 10.3390/jof8101109

**Published:** 2022-10-20

**Authors:** Laurent Dufossé

**Affiliations:** CHEMBIOPRO Laboratoire de Chimie et Biotechnologie des Produits Naturels, ESIROI Agroalimentaire, Université de La Réunion, 15 Avenue René Cassin, F-97400 Saint-Denis, Ile de La Réunion, France; laurent.dufosse@univ-reunion.fr; Tel.: +33-262-692-402-400

Following the previous Journal of Fungi (ISSN 2309-608X) Fungal Pigments Special Issue edited and published in 2017 (weblink https://www.mdpi.com/journal/jof/special_issues/fungal_pigments), with 10 papers and the Fungal Pigments printed book edition of 134 pages (weblink https://www.mdpi.com/books/book/570), the time has come to open a new edition entitled Fungal Pigments 2021 which presents the latest scientific advances in this field from August 2020 to August 2021.

With the impact of globalization in research trends, the search for healthier lifestyles, the increasing public demand for natural, organic, and “clean labelled” products, as well as the growing global market for natural colorants in economically fast-growing countries all over the world, filamentous fungi started to be investigated as readily available sources of chemically diverse pigments and colorants. For all of these reasons, this new Special Issue of the Journal of Fungi intends to highlight exciting findings, which may pave the way for alternative and/or additional biotechnological processes for industrial applications of fungal pigments and colorants. Research papers and reviews on fungal biodiversity from terrestrial and marine origins were welcomed, thus contributing new findings on fungi as potential sources of well-known carotenoid pigments (e.g., beta-carotene, lycopene) and other specific pigmented polyketide molecules, such as *Monascus* and *Monascus*-like azaphilones, which are not yet known to be biosynthesized by any other organisms such as higher plants. These polyketide pigments also include promising and unexplored hydroxy-anthraquinoid colorants from Ascomycetous species. The investigation into the biosynthetic pathways of the carotenoids and polyketide-derivative colored molecules (i.e., azaphilones, hydroxyanthraquinones, and naphthoquinones) in pigment-producing fungal species contribute to the additional articles. Contributions on alternative greener extraction processes of fungal colored compounds, along with current industrial applications, descriptions of their limits, and further opportunities for the use of fungal pigments in beverage, food, pharmaceutical, cosmetic, textile, and painting areas are also projected to be part of this Special Issue.

All these subjects and more are covered by the 11 articles published in this Special Issue: 

weblink https://www.mdpi.com/journal/jof/special_issues/fungal_pigments_2021.

* *Investigation on various chemical classes of fungal pigments (melanins, azaphilones, quinones, etc.)*

**Genomic Analysis and Assessment of Melanin Synthesis in *Amorphotheca resinae* KUC3009** by Jeong-Joo Oh et al.; https://doi.org/10.3390/jof7040289.
**Fungal Melanins and Applications in Healthcare, Bioremediation and Industry** by Ellie Rose Mattoon et al.; https://doi.org/10.3390/jof7060488.
**RecentFindings in Azaphilone Pigments** by Lúcia P. S. Pimenta et al.; https://doi.org/10.3390/jof7070541.
**Characterization of a Biofilm Bioreactor Designed for the Single-Step Production of Aerial Conidia and Oosporein** by *Beauveria bassiana* PQ2 by Héctor Raziel Lara-Juache et al.; https://doi.org/10.3390/jof7080582.

* *Molecular characterization*

**MolecularCharacterization of Fungal Pigments** by Miriam S. Valenzuela-Gloria et al.; https://doi.org/10.3390/jof7050326.

* *Biological properties*

**Seven NewCytotoxic and Antimicrobial Xanthoquinodins from *Jugulospora vestita*** by Lulu Shao et al.; https://doi.org/10.3390/jof6040188.

* *Toxicity assessment and safety evaluation of fungal pigments*

**SafetyEvaluation of Fungal Pigments for Food Applications** by Rajendran Poorniammal et al.; https://doi.org/10.3390/jof7090692.
**Preliminary Examination of the Toxicity of Spalting Fungal Pigments: A Comparison between Extraction Methods** by Badria H. Almurshidi et al.; https://doi.org/10.3390/jof7020155.

* *Use of by-products or waste for industrial production of fungal pigments*

**Production of Bio-Based Pigments from Food Processing Industry By-Products (Apple, Pomegranate, Black Carrot, Red Beet Pulps) Using *Aspergillus carbonarius*** by Ezgi Bezirhan Arikan et al.; https://doi.org/10.3390/jof6040240.

* *Prospective aspects and brainstorming*

**DoesStructural Color Exist in True Fungi?** by Juliet Brodie et al.; https://doi.org/10.3390/jof7020141.
**FungalBiomarkers Stability in Mars Regolith Analogues after Simulated Space and Mars-like Conditions** by Alessia Cassaro et al.; https://doi.org/10.3390/jof7100859.

I, as Guest Editor, trust all readers of this Special Issue enjoy the contents and I would like to deeply thank all 62 authors who contributed (sorted by their last names), Prof. Dr. David S. Perlin, Editor-in-Chief of the Journal of Fungi, the numerous reviewers, and the whole team at MDPI (editing, production, website, etc.):
Aguilar, Cristóbal N.Dufossé, LaurentPaul de Vera, Jean-PierreAguilar, OscarGomes, Dhionne C.Pimenta, Lúcia P.S.Aguilar-Zárate, MayraHarper, BryanPoorniammal, RajendranAguilar-Zárate, PedroHarper, StaceyPrabhu, SomasundaramAlmurshidi, Badria H.Hernández-Almanza, AyerimRabbow, ElkeArikan, Ezgi BezirhanIngham, Colin J.Ramona Michel, MarielaAscacio-Valdés, Juan AlbertoKannan, JegatheeshRobinson, Seri C.Ávila-Hernández, José GuadalupeKim, Gyu-HyeokRodríguez-Durá, Luis VíctorBalagurusamy, NagamaniKim, Jee YoungSaladino, RaffaeleBaqué, MickaelKim, Jung WooShao, LuluBotta, LorenzoKim, Young JunStadler, MarcBöttger, UteKwon, Sun LulStchigel, Alberto M.Brodie, JulietLara-Juache, Héctor RazielSurup, FrankCanli, OltanLee, ChangsuTakahashi, Jacqueline A.Cardoso, Patrícia G.Lee, Myeong-EunValenzuela-Gloria, Miriam S.Caro, YanisMarin-Felix, YasminaVan Court, R.C.Casadevall, ArturoMattoon, Ellie RoseVeana, FabiolaCassaro, AlessiaMuñiz-Márquez, Diana BeatrizVega Gutierrez, Sarath M.Chávez-González, Mónica L.Oh, Jeong-JooVignolini, SilviaCordero, Radames J.B.Onofri, SilvanoWong-Paz, Jorge EnriqueDizge, NadirPacelli, Claudia


